# Multidrug-resistant *Acinetobacter baumannii* infections in COVID-19 patients hospitalized in intensive care unit

**DOI:** 10.1007/s15010-021-01643-4

**Published:** 2021-06-27

**Authors:** Alessandro Russo, Francesca Gavaruzzi, Giancarlo Ceccarelli, Cristian Borrazzo, Alessandra Oliva, Francesco Alessandri, Eugenia Magnanimi, Francesco Pugliese, Mario Venditti

**Affiliations:** 1 Infectious and Tropical Diseases Unit, Department of Medical and Surgical Sciences, “Magna Graecia” University of Catanzaro, Catanzaro, Italy; 2 Department of Public Health and Infectious Diseases, “Sapienza” University of Rome, Viale dell’Università 37, 00161 Rome, Italy; 3 Department of Anesthesia and Critical Care Medicine, “Sapienza” University of Rome, Rome, Italy

**Keywords:** *Acinetobacter baumannii*, COVID-19, Bacteraemia, Colonization, Steroids

## Abstract

**Objectives:**

Superinfections in patients hospitalized in intensive care unit (ICU) are an important and challenging complication, also in COVID-19. However, no definitive data are available about the role of multidrug-resistant *Acinetobacter baumannii* (MDR-AB) in COVID-19.

**Methods:**

This was a single-center, cross-sectional study including patients with MDR-AB infections admitted to ICU with or without COVID-19, between January 2019 and January 2021. The primary objective of the study was to evaluate risk factor for MDR-AB infections in ICU patients hospitalized for COVID-19 or other etiology. The secondary endpoints were 30-days mortality in all study population and risk factors associated with development of bloodstream infection (BSI).

**Results:**

During the study period 32 adults with COVID-19 were enrolled and compared with 115 patients admitted in the same ICU for other reasons. We observed a total of 114 deaths, with a survival rate of 29.3%: 18.8% in COVID-19 and 32.2% in control group. Relative risk for MDR-AB infection in COVID-19 showed that serum lactate levels mmol/l > 2, *Acinetobacter baumannii* colonization, BSI and steroid therapy were observed more frequently in COVID-19 patients. Cox regression analysis showed that serum lactate levels > 2 mmol/l, *Acinetobacter baumannii* colonization, BSI, and steroid therapy were associated with 30-days mortality. Finally, patients with COVID-19, white blood cells count > 11,000 mm^3^, serum lactate levels > 2 mmol/l, infections at time of ICU admission, *Acinetobacter baumannii* colonization, and steroid therapy were independently associated with development of BSI.

**Conclusions:**

Our data highlight the impact of BSI on outcome, the role of *Acinetobacter baumannii* colonization and the use of steroids on the risk to develop MDR-AB infections also during COVID-19.

## Introduction

Since the end of 2019 the Coronavirus Disease 2019 (COVID-19), caused by severe acute respiratory syndrome coronavirus 2 (SARS-CoV-2), has spread globally affecting people worldwide [1, 2]. Patients with severe COVID-19 require intensive care unit (ICU) admission for acute respiratory failure and over 10% need noninvasive and invasive mechanical ventilation [3, 4]. Acute respiratory distress syndrome (ARDS) severity and ventilation management determine a negative outcome and a 90-days mortality of 31% [2].

The data about superinfections complicating COVID-19 are scant, and a significant proportion of these patients are treated with empiric broad spectrum antibiotic therapy that increase the risk to develop infections caused by multidrug-resistant (MDR) pathogens [5, 6]. Finally, the use of drugs targeting cytokines, such as IL-1 and IL-6, might also increase the risk of superinfections in patients with COVID-19 [7].

Infections caused by MDR *Acinetobacter baumannii* (MDR-AB) represent a major problem in patients admitted to the intensive care unit (ICU) [8, 9]. Inappropriate therapy and limited therapeutic options are responsible for negative impact on outcome and this infection is associated with high mortality rates, especially in ICU patients [10, 11].

The aim of our study was to evaluate the impact of MDR-AB infections on outcome of patients with COVID-19 requiring ICU admission, comparing with non-COVID-19 patients with MDR-AB infections hospitalized in the same ward. We evaluated risk factor for acquisition of MDR-AB infections in ICU patients hospitalized for COVID-19 or other etiology, 30-days mortality in all study population, and risk factors associated with development of bloodstream infection (BSI).

## Methods

### Study design and patient selection

This was a single-center, cross-sectional study including patients with MDR-AB infections consecutively admitted to the tertiary care Policlinico Umberto I, Sapienza University of Rome, Italy, between January 2019 and January 2021. We compare patients divided in two groups: patients with and without COVID-19 admitted to ICU. Inclusion criteria for all patients were: (1) age ≥ 18 years; (2) clinical signs and symptoms consistent with infection; (3) documented MDR-AB etiology. All patients were managed by the same team of physicians and all antimicrobial therapies were selected according to clinical judgment by infectious disease specialists. The prospective nature of the study was based on the consecutive enrollment of patients. However, all complete data were afterwards retrospectively extracted, and the local Ethics Committee waived the need for informed consent. The study was conducted according to the principles stated in the Declaration of Helsinki.

Patients’ data were collected from medical charts and from hospital computerized databases. The following information were reviewed: demographics; clinical, and laboratory findings; comorbid conditions and the age-adjusted Charlson Comorbidity Index; microbiological data; duration of ICU and hospital stay; any infection during hospitalization; duration of antibiotic therapy, and use of steroid therapy; procedures (e.g., mechanical ventilation, continuous renal replacement therapy [CRRT]), extracorporeal membrane oxygenation [ECMO], carried out during hospitalization; the simplified acute physiology score (SAPS II); sequential organ failure assessment (SOFA) and quick (q)-SOFA, serum lactate levels mmol > 2 at time of infection; anamnestic MDR-AB colonization or infection during hospitalization; source of infection and its adequate control; development of bacteremias and septic shock; 30-days mortality.

### Definitions

Septic shock was defined according to international definitions [12].

The severity of clinical conditions was determined using SAPS II, SOFA, and qSOFA scores calculated at the time of infection onset.

The length of hospital and ICU stay were calculated as the number of days from the date of admission to the date of discharge or death. Adequate control of source of infection was defined as the removal of any preexisting contaminated CVC as well as the drainage of intra-abdominal abscesses or other fluid collections have been performed within 24 h after the onset of infection. The timing of CVC removal was based on the medical record review, and was confirmed by review of patient radiographs.

MDR-AB infections were classified in the following categories: ventilator-associated pneumonia (VAP), hospital-acquired pneumonia (HAP), urinary tract infection (UTI), and BSI. *Acinetobacter baumannii* colonization was weekly evaluated with an active surveillance in hospitalized patients.

Inclusion criteria for patients with COVID-19 were: (1) laboratory confirmed SARS-CoV-2 infection with an RT–PCR test on a nasopharyngeal swab; (2) uni- or bi-lateral interstitial infiltrates confirmed by CT scan or chest X ray; (3) presence of acute hypoxemic respiratory failure requiring mechanical ventilation.

All of these clinically indicated infections were categorized as co-infections or superinfections. If diagnosis was at the time of or within the first 24 h of COVID-19 hospital admission, these infections were defined as community-acquired co-infections. If diagnosis occurred ≥ 48 h after admission for COVID-19, these infections were defined as hospital-acquired superinfections.

### MDR-AB definition

Identification of MDR-AB strains was based accordingly with local laboratory techniques. The Vitek 2 automated system (bioMérieux, Marcy l’Etoile, France) was used for isolate identification and antimicrobial susceptibility testing. Minimum inhibitory concentrations (MICs) were established according to the European Committee on Antimicrobial Susceptibility Testing (EU- CAST) breakpoints [13]. Isolated strains were classified as multidrug resistant (MDR), extensively drug resistant (XDR), and pandrug resistant (PDR) [14].

### Primary endpoint and statistical analysis

The primary objective of the study was to evaluate risk factor for MDR-AB infections in ICU patients hospitalized for COVID-19 or other etiology. The secondary endpoints were 30-days mortality in all study population and risk factors associated with development of BSI.

All data were analyzed using Statistical Package for Social Science (SPSS) version 20 or Microsoft Excel (Office 2018). Description of mean ± standard deviation (SD), simple frequencies (*n*), proportions (or percentages), and rates of the given data on each variable has been calculated. The univariate analysis was used to compare patients divided in two groups: MDR-AB infection in patient with COVID-19 vs No COVID-19. T test was conducted for continuous variables and chi-square for categorical variables. The odds ratio (OR) and 95% confidence intervals (CI) were used to quantify the strength of the association between covariates and dependent variable. We have done a standard survival analysis, tracing participants affected, or not by COVID-19 from entry into the clinic to the discharge or death at 30 days. The event‐free survival in follow‐up was depicted graphically by Kaplan–Meier’s survival curve, including the confounding factors with fixed baseline covariates. A *p* value of less than 0.05 was considered statistically significant.

## Results

**Fig. 1:**
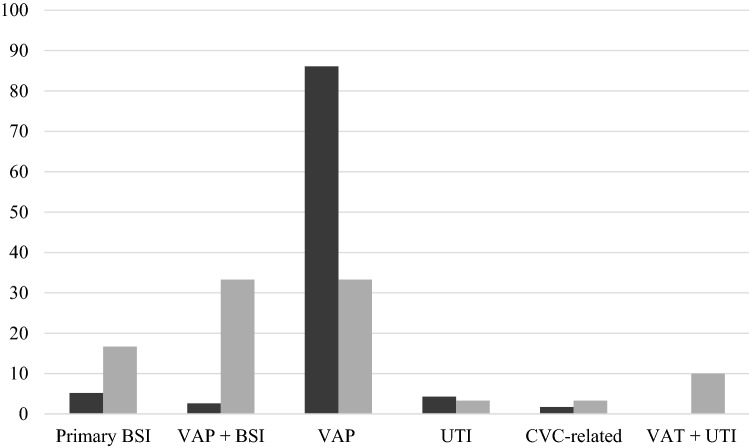
Sites of MDR-AB infection in COVID-19 (gray line) or non-COVID-19 (black line). *MDR-AB* multidrug-resistant *Acinetobacter baumannii*, *BSI* bloodstream infection, *VAP* ventilator-associated pneumonia, *UTI* urinary tract infection, *CVC* central venous catheter, *VAT* ventilator-associated tracheobronchitis

During the study period, 32 adults with COVID-19 and superinfection caused by MDR-AB were prospectively enrolled. This cohort of patients was compared with 115 patients with MDR-AB infection admitted in the same ICU for other etiologies: respiratory failure (29%), septic shock (26%), trauma (20%), stroke (15%), cardiac/hemorrhagic shock/postsurgery (10%). Overall, 147 patients were evaluated in the final analysis. Sites of MDR-AB infection in study population are reported in Fig. 1.

Table 1 shows univariate analysis comparing clinical characteristics of patients affected or not by COVID-19 with documented MDR-AB infection. Differences between COVID-19 and other patients were reported for previous hospitalization (16% vs. 39%, *p* < 0.015), chronic kidney disease (3% vs. 19%, *p* < 0.001), COPD (9% vs. 32%, *p* < 0.009), chronic corticosteroid therapy (0% vs. 27%, *p* < 0.001), and previous *Acinetobacter baumannii* colonization or infection (0% vs. 15%, *p* < 0.020). No differences were observed between COVID-19 and other patients related to the median age, Charlson Comorbidity Index, length of hospitalization, and ICU stay, SAPS II at time of admission, procedures (e.g., mechanical ventilation), clinical and laboratory findings at time of infection, duration of antibiotic therapy, development of septic shock, 30-days mortality.

**Table 1:** Univariate analysis about clinical characteristics and outcome of patients with MDR-AB infections affected or not by COVID-19

Variables	COVID-19 *n* = 32 (%)	Non-COVID-19 *n* = 115 (%)	*p* value
***Anamnestic factors and comorbidities***			
Male sex	21 (66%)	73 (63%)	0.543
Age, mean ± SD (years)	62.50 ± 10.99	62.59 ± 11.31	0.969
Previous hospitalization (90 days)	5 (16%)	45 (39%)	**0.015**
Previous ICU admission (90 days)	3 (9%)	22 (19%)	0.132
> 2 comorbidities	11 (34%)	41 (36%)	0.895
Cardiovascular disease	17 (53%)	62 (54%)	0.938
Heart failure	3 (9%)	9 (8%)	0.791
Charlson Comorbidity Index, mean ± SD	2.59 ± 1.81	2.79 ± 1.78	0.605
Diabetes	4 (13%)	12 (10%)	0.756
Chronic kidney disease	1 (3%)	22 (19%)	**0.001**
Chronic liver disease	1 (3%)	8 (7%)	0.333
Neurologic disease	3 (9%)	19 (17%)	0.267
Vasculitis	1 (3%)	9 (8%)	0.245
COPD	3 (9%)	37 (32%)	**0.009**
Solid tumor	2 (6%)	9 (8%)	0.755
Hematological malignancies	1 (3%)	8 (7%)	0.333
Chronic corticosteroid therapy	0	31 (27%)	**0.001**
Previous *Acinetobacter baumannii* infection (30 days)	0	17 (15%)	**0.020**
Previous endoscopy procedure (30 days)	3 (9%)	16 (14%)	0.464
Intravascular devices	5 (16%)	17 (15%)	0.909
Previous antibiotic therapy (30 days)	9 (28%)	45 (39%)	0.241
***Clinical features***			
SAPS II at time of admission, mean ± SD	33.75 ± 15.57	37.10 ± 17.75	0.296
GCS at the time of admission, mean ± SD	14.65 ± 0.00	15.00 ± 0.00	0.330
PaO_2_/FiO_2_ < 250	26 (81%)	88 (77%)	0.560
WBC, mean ± SD	10,937.81 ± 8794.10	13,411.83 ± 12,375.35	0.209
Surgery	2 (6%)	9 (8%)	0.755
PLTs, mean ± SD	254,656.25 ± 101,504.71	246,200.00 ± 88,537.26	0.679
SOFA at the time of admission, mean ± SD	5.31 ± 2.95	4.92 ± 2.55	0.487
Quick SOFA at the time of admission, mean ± SD	0.72 ± 0.70	0.83 ± 0.81	0.454
CRRT	13 (41%)	46 (40%)	0.949
ECMO	4 (13%)	15 (13%)	1.000
Surgery source control	3 (9%)	20 (17%)	0.267
Septic shock	8 (25%)	43 (37%)	0.208
SOFA at time of infection onset, mean ± SD	8.31 ± 4.29	7.48 ± 3.76	0.312
Quick SOFA at time of infection onset, mean ± SD	1.25 ± 0.90	1.28 ± 0.93	0.875
PCT at time of infection onset, mean ± SD	4.01 ± 6.33	3.73 ± 5.23	0.825
Serum lactate levels > 2 mmol/l	21 (66%)	42 (37%)	**0.003**
MDR colonization at the time of ICU admission	1 (3%)	16 (14%)	0.068
Infections at time of ICU admission	2 (6%)	70 (61%)	** < 0.001**
*Acinetobacter baumannii* colonization	20 (63%)	9 (8%)	** < 0.001**
Time from colonization to MDR-AB infection, mean ± SD (days)	10.21 ± 9.85	11.82 ± 9.2	0.89
Bloodstream infection	18 (56%)	9 (8%)	** < 0.001**
***Outcomes and therapy***			
Steroid therapy	28 (88%)	5 (4%)	** < 0.001**
Total duration of antibiotic therapy, mean ± SD	25.56 ± 12.66	25.35 ± 14.87	0.936
Transfer in ICU	31 (97%)	115 (100%)	0.325
Length of ICU stay, mean ± SD (days)	22.22 ± 9.65	22.23 ± 9.53	0.997
Length of hospitalization, mean ± SD (days)	30.41 ± 13.56	29.10 ± 10.59	0.610
Mortality at 30 days	26 (81%)	78 (68%)	0.154

Statistically significant *p*-values are in bold

*MDR-AB* multidrug-resistant *Acinetobacter baumannii*, *SD* standard deviation, *ICU* intensive care unit, *COPD* chronic obstructive pulmonary disease, *SAPS* simplified acute physiology score, *GCS* Glasgow coma score, *WBC* white blood cell, *PLT* platelets, *SOFA* sequential organ failure assessment, *PICC* peripherally-inserted central catheter, *CVC* central venous catheter, *CRRT* continuous renal replacement therapy, *ECMO* extracorporeal membrane oxygenation, *PCT* procalcitonin, *CRP* C-reactive protein

Relative risk associated with COVID-19 vs. non-COVID-19 etiology was reported in Table 2: previous hospitalization (RR 0.4; CI 95% 0.2–0.9, *p* = 0.031), COPD (RR 0.3, CI 95% 0.1–0.9, *p* = 0.029), chronic corticosteroid therapy (RR 0.1, CI 95% 0.0–0.9, *p* = 0.041) and infection at time of ICU admission (RR 0.1, CI 95% 0.0–0.9, *p* = 0.001) were factors associated with non-COVID-19 etiology. Conversely, serum lactate levels > 2 mmol/l at time of infection (RR 1.8, CI 95% 1.3–2.5, *p* = 0.001), *Acinetobacter baumannii* colonization (RR 7.9, CI 95% 4.0–15.7, *p* < 0.001), BSI (RR 6.5, CI 95% 3.2–13.3, *p* < 0.001), and steroid therapy (RR 18.4, CI 95% 7.6–44.1, *p* < 0.001) were observed more frequently in COVID-19 patients.

**Table 2:** Relative risk* associated or not with MDR-AB infection in patients affected or not by COVID-19

Variables	RR	CI 95%	*p* value
Previous hospitalization (90 days)	0.4	0.2–0.9	0.031
COPD	0.3	0.1–0.9	0.029
Chronic steroid therapy	0.1	0.0–0.9	0.041
Infection at time of ICU admission	0.1	0.0–0.4	0.001
Serum lactate levels > 2 mmol/l	1.8	1.3–2.5	0.001
*Acinetobacter baumannii* colonization	7.9	4.0–15.7	< 0.001
Bloodstream infection	6.5	3.2–13.3	< 0.001
Steroid therapy	18.4	7.6–44.1	< 0.001

*RR* relative risk, *CI* confidence interval, *COPD* chronic obstructive pulmonary disease, *ICU* intensive care unit

*RR < 1 is associated with non-COVID-19 etiology; > 1 with COVID-19

Cox regression analysis of factors associated with 30-days mortality (see Table 3) showed that serum lactate levels > 2 mmol/l at time of infection (OR 4.9; CI 95% 2.1–11.3, *p* < 0.001), *Acinetobacter baumannii* colonization (OR 17.1, CI 95% 5.5–53.3, *p* < 0.001), BSI (OR 13.6; CI 95% 4.8–38.2, *p* < 0.001) and steroid therapy (OR 46.9; CI 95% 13.9–157.3, *p* < 0.001) were associated with 30-days mortality.

**Table 3:** Logistic regression analysis about risk factors associated with 30-days mortality

Variables	OR	CI 95%	*p* value
Serum lactate levels > 2 mmol/l	4.9	2.1–11.3	< 0.001
*Acinetobacter baumannii* colonization	17.1	5.5–53.3	< 0.001
Bloodstream infection	13.6	4.8–38.2	< 0.001
Steroid therapy	46.9	13.9–157.5	< 0.001

*OR* odds ratio, *CI* confidence interval

**Fig. 2:**
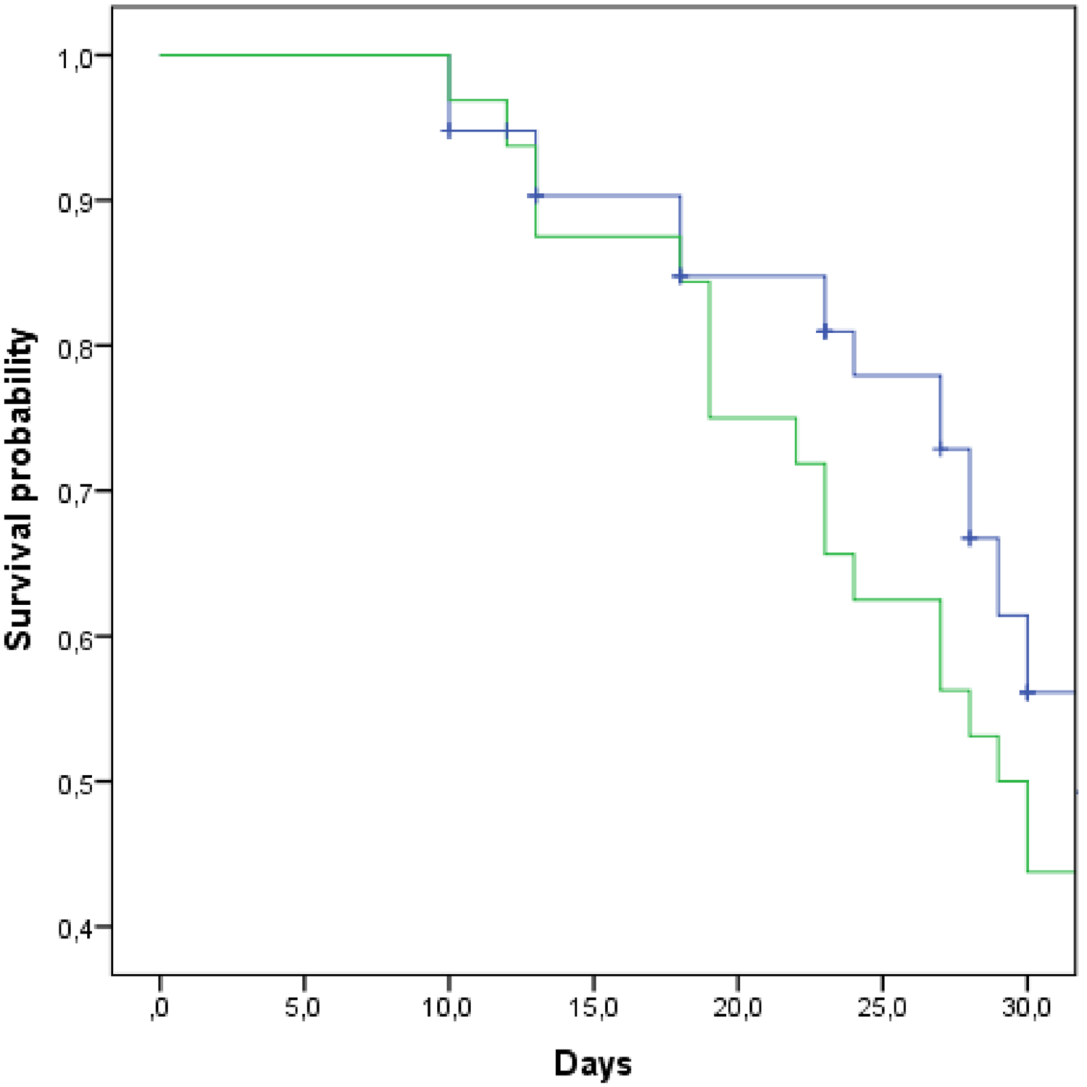
Kaplan–Meier curves for 30-days survival in patients affected (green line) or not (blue line) by COVID-19

The Kaplan–Meier curves for 30-days survival of overall patients with MDR-AB infections (COVID-19 or non-COVID-19 etiology) is reported in Fig. 2. We observed a total of 114 deaths, with a survival rate of 29.3%. Comparing the two groups, we observed a different survival rate: 18.8% (COVID-19) and 32.2% (other patients).

Table 4 shows univariate analysis comparing patients developing or not BSI caused by MDR-AB. COVID-19 etiology (67% vs. 12%, *p* < 0.001), serum lactate levels > 2 mmol/l at time of infection (63% vs. 38%, *p* = 0.018), *Acinetobacter baumannii* colonization (44% vs. 14%, *p* = 0.006) and a steroid therapy during the hospitalization (63% vs. 13%, *p* < 0.001) were more frequently reported in patients developing MDR-AB BSI.

**Table 4:** Univariate analysis comparing patients developing or not bloodstream infection

Variables	No bloodstream infection *n* = 120 (%)	Bloodstream infection *n* = 27 (%)	*p* value
***Anamnestic factors and comorbidities***			
COVID-19	14 (12%)	18 (67%)	** < 0.001**
Male sex	77 (64%),	17 (63%)	0.922
Age, mean ± SD (years)	61.84 ± 11.55	65.81 ± 9.86	0.074
Previous hospitalization (90 days)	42 (35%)	8 (30%)	0.593
Previous ICU admission (90 days)	22 (18%)	3 (11%)	0.315
> 2 comorbidities	43 (36%)	9 (33%)	0.808
Cardiovascular disease	67 (56%)	12 (44%)	0.260
Heart failure	10 (8%)	2 (7%)	0.872
Charlson Comorbidity Index mean ± SD	2.73 ± 1.78	2.85 ± 1.97	0.761
Diabetes	13 (11%)	3 (11%)	1.000
Chronic kidney disease	18 (15%)	5 (19%)	0.674
Chronic liver disease	9 (8%)	0 (0%)	0.129
Neurologic disease	17 (14%)	5 (19%)	0.602
Vasculitis	8 (7%)	2 (7%)	1.000
COPD	34 (28%)	6 (22%)	0.508
Solid tumor	10 (8%)	1 (4%)	0.307
Hematological malignancies	9 (8%)	0	0.129
Chronic corticosteroid therapy	27 (23%)	4 (15%)	0.339
Previous *Acinetobacter baumannii* infection (30 days)	16 (13%)	1 (4%)	0.051
Previous endoscopy procedure (30 days)	18 (15%)	1 (4%)	0.157
Intravascular devices	16 (13%)	6 (22%)	0.316
Previous antibiotic therapy (30 days)	47 (39%)	7 (26%)	0.179
***Clinical features***			
SAPS II at time of admission, mean ± SD	37.01 ± 17.69	33.56 ± 15.24	0.308
GCS at the time of admission, mean ± SD	15.00 ± 0.00	14.63 ± 1.61	0.331
PaO2/FiO2 < 250	95 (79%)	19 (70%)	0.207
WBC mean ± SD	13,707.75 ± 12,658.93	9164.44 ± 4563.52	**0.002**
Surgery	10 (8%)	1 (4%)	0.307
PTL mean ± SD	248,166.67 ± 88,895.02	247,481.48 ± 106,697.40	0.975
SOFA at the time of admission, mean ± SD	5.02 ± 2.59	4.96 ± 2.79	0.928
Quick SOFA at the time of admission, mean ± SD	0.83 ± 0.81	0.67 ± 0.62	0.241
CRRT	45 (37.5)	14 (51.8)	0.06
ECMO	18 (15%)	1 (4%)	0.157
Surgery source control	22 (18%)	1 (4%)	0.069
Septic shock	40 (33.3)	11 (40.7)	0.384
SOFA at time of infection onset, mean ± SD	7.44 ± 3.72	8.63 ± 4.33	0.196
qSOFA at time of infection onset, mean ± SD	1.24 ± 0.92	1.41 ± 0.93	0.407
PCT at time of infection onset, mean ± SD	3.75 ± 5.52	3.97 ± 5.18	0.847
Serum lactate levels > 2 mmol/l	46 (38%)	17 (63%)	**0.018**
MDR colonization at the time of ICU admission	15 (13%)	2 (7%)	0.386
Infection at time of ICU admission	64 (53%)	8 (30%)	**0.031**
*Acinetobacter baumannii* colonization	17 (14%)	12 (44%)	**0.006**
Time from colonization to MDR-AB infection, mean ± SD (days)	13.34 ± 11.81	10.91 ± 8.2	**0.03**
***Outcomes and therapy***			
Steroid therapy	16 (13%)	17 (63%)	** < 0.001**
Total duration of antibiotic therapy, mean ± SD	25.83 ± 15.19	23.44 ± 10.48	0.333
Transfer in ICU	120 (100%)	26 (96%)	0.327
Length of ICU stay, mean ± SD (days)	21.75 ± 9.17	24.33 ± 11.51	0.283
Length of hospitalization, mean ± SD (days)	29.46 ± 11.20	29.07 ± 11.25	0.873
Mortality at 30 days	84 (70%)	20 (74%)	0.673

Statistically significant *p*-values are in bold

*MDR-AB* multidrug-resistant *Acinetobacter baumannii*, *SD* standard deviation, *ICU* intensive care unit, *COPD* chronic obstructive pulmonary disease, *SAPS* simplified acute physiology score, *GCS* Glasgow coma score, *WBC* white blood cell, *PLT* platelets, *SOFA* sequential organ failure assessment, *PICC* peripherally-inserted central catheter, *CVC* central venous catheter, *CRRT* continuous renal replacement therapy, *ECMO* extracorporeal membrane oxygenation, *PCT* procalcitonin, *CRP* C-reactive protein

Finally, multivariate analysis about risk factors associated with development of BSI (see Table 5) showed that patients with COVID-19 (OR 15.1, CI 95% 3.7–40.1; *p* < 0.001), white blood cells count > 11,000 mm^3^ (OR 5.2, CI 95% 2.1–11.5; *p* < 0.001), serum lactate levels > 2 mmol/l (OR 2.7; CI 95% 1.2–6.4, *p* = 0.022), infections at time of ICU admission (OR 0.4, CI 95% 0.2–1.0, *p* = 0.030), *Acinetobacter baumannii* colonization (OR 4.8, CI 95% 1.9–12.1, *p* < 0.001), and steroid therapy during hospitalization (OR 8.8, CI 95% 3.5–22.1, *p* < 0.001) were factors independently associated with development of BSI.

**Table 5:** Multivariate analysis about risk factors associated with development of bloodstream infection

Variables	OR	CI 95%	*p* value
Severe COVID-19	15.1	3.7–40.1	< 0.001
WBC > 11,000 mm^3^	5.2	2.1–11.5	< 0.001
Serum lactate levels > 2 mmol/l	2.7	1.2–6.4	0.022
Infections at time of ICU admission	0.4	0.2–1	0.030
*Acinetobacter baumannii* colonization	4.8	1.9–12.1	< 0.001
Steroid therapy	8.8	3.5–22.1	< 0.001

*OR* odds ratio, *CI* confidence interval, *WBC* white blood cell, *ICU* intensive care unit

## Discussion

To our knowledge, this is the largest experience about risk factors and outcomes of MDR-AB infections in patients affected or not by COVID-19 in ICU. Our study confirms that bacterial superinfections may complicate the hospital course of patients with COVID-19, and we identified peculiar characteristics of COVID-19 patients developing these difficult-to-treat infections. Our data showed that serum lactate levels > 2 mmol/l at time of infection, *Acinetobacter baumannii* colonization, development of a BSI, and steroid therapy were the most important factors associated with MDR-AB infection in COVID-19 patients and resulted as important determinants of 30-day mortality also in all study population.

Moreover, COVID-19 etiology, white blood cells count > 11,000 mm^3^, serum lactate levels > 2 mmol/l, infection at time of ICU admission, *Acinetobacter baumannii* colonization, and steroid therapy during hospitalization were associated with higher risk of BSI development. In this study, our data showed as MDR-AB BSI remain an important ICU-acquired infection [8, 9, 11–15].

Of interest, our experience highlighted the importance of superinfections caused by Gram-negative strains in ICU, including COVID-19 patients. Of importance, a rapid spread of MDR gram-negative bacteria among patients in dedicated coronavirus disease care units was recently observed [16]. In a recent meta-analysis, 19% of patients with COVID-19 showed co-infections and 24% superinfections; the presence of either co-infection or superinfection was associated with poor outcomes, including increased mortality [17].

First of all, this data reflect local epidemiology characterized by a high prevalence of MDR Gram-negatives. The data from literature suggest that COVID-19 was associated with a less effective implementation of infection control procedures for several reasons [18]. As a matter of fact, health-care workers (HCWs) experimented important difficulties to apply standard precautions, and to wear the same equipment for a long time; moreover, HCWs mainly focused on self-protection rather than on cross-transmission of bacteria in the wards. Finally, overcrowded wards, shortages of professionals with appropriate training in infection control procedures, and possible decreased laboratory ability to detect MDR carriage are potentially considered risk factors of MDR spread after the COVID-19 outbreak [19, 20]. Then, it will be crucial to continue monitoring rates of MDR infections and implementing measures of infection control and antimicrobial stewardship [21–23].

Of note, *Acinetobacter baumannii* intestinal colonization resulted as an independent predictor of infection in COVID-19 patients. As a matter of fact, the association between rectal carriage by carbapenem-resistant pathogens and development of infection is reported as an important predictor of infection, especially in ICU patients [24]. There are many important observations about the role of the gut microbiota during SARS-CoV-2 infection. The gut microbiota of COVID-19 patients is characterized by enrichment of opportunistic pathogens and alterations in gut cells also in the absence of gastrointestinal manifestations [19, 25, 26]. Of interest, these alterations were observed after hospitalization; however, the administration of broad-spectrum antibiotics is the major determinant for intestinal colonization by MDR pathogens.

Our data showed that MDR-AB BSI remains an important ICU-acquired infection, associated with higher mortality. In our interpretation, sepsis and septic shock determine a lethal cascade of events that is unlikely to be interrupted even by an appropriate initial antimicrobial treatment. In addition, most of our patients were severely ill and would probably have been unable to survive their infections independently of the administration of an adequate initial antimicrobial treatment. The data about the high rate of unfavorable outcome in patient with MDR-AB BSI were previously reported and discussed [8, 9, 11]. Finally, MDR-AB can be considered as a marker of the severity for the underlying diseases.

A peculiar aspect of COVID-19 patients, especially during the “second wave”, was the widely use of steroids at high dosages. The use of dexamethasone resulted in lower 28-day mortality, especially in patients receiving invasive mechanical ventilation [27, 28]. However, the prolonged use of high doses of steroids could be associated with the well-known immunomodulant effects of these drugs [29], but the association between steroid treatment and MDR infections deserves further comments. We can hypothesize that patients who received steroids survived longer and were, therefore, more likely to develop an MDR infection during ICU stay. However, in COVID-19 patients with a prolonged ICU stay the use of steroids and immunomodulant drugs may increase the risk of superinfections and should be used with caution [30, 31].

Our study has some limitations. First, it is a single-center study conducted in a setting with a high prevalence of MDR pathogens. Second, the sample size is relatively small, and the CIs of some significant predictors are quite broad. Third, we did not perform a multilocus sequence typing of the strains to understand if we observed an outbreak of infection. Finally, the impact of some therapies, including immunomodulant drugs, and of empiric and definitive antibiotic regimens for treatment of MDR-AB infections were not definitively assessed in the final analysis.

In conclusion, we reported a single-center experience about MDR-AB infection in COVID-19 patients, comparing those with ICU patients hospitalized for other etiologies. Our data highlight the impact of BSI on outcome, the role of *Acinetobacter baumannii* colonization and the use of steroids on the risk to develop MDR-AB infections also during COVID-19. Antimicrobial stewardship programs are mandatory in this population [32, 33].

## Declarations

### Transparency declarations

None to declare.
